# Effect of Nitric Oxide on Human Corneal Epithelial Cell Viability and Corneal Wound Healing

**DOI:** 10.1038/s41598-017-08576-9

**Published:** 2017-08-14

**Authors:** Joo-Hee Park, Ja-Yeon Kim, Dong Ju Kim, Martha Kim, Minwook Chang, Roy S. Chuck, Choul Yong Park

**Affiliations:** 1 Department of Ophthalmology, Dongguk University, Ilsan Hospital, Goyang, South Korea; 2 Department of Ophthalmology and Visual Sciences, Montefiore Medical Center, Albert Einstein College of Medicine, Bronx, NY USA

## Abstract

Although the wound healing effects of nitric oxide (NO) are known, the mechanism by which NO modulates corneal wound healing remains unclear. In this study, we investigated the effect of exogenous NO donor (NaNO_2_) on corneal wound healing. We found that NaNO_2_ (0.1 μM to 100 μM) increased human corneal epithelial cell (HCEC) viability and migration. It also modulated the phosphorylation of mitogen-activated protein kinases (MAPKs) in a time- dependent manner in those HCECs. Further, p38 MAPK phosphorylation increased at 6 h and normalized at 24 h, while the phosphorylation of extracellular signal regulated kinase (ERK) was increased both at 6 h and 24 h. Topical treatment with NaNO_2_ (10 μM) enhanced corneal epithelial healing and decreased corneal opacity in murine corneal alkali burn model by modulating inflammatory cytokines. Our findings suggest that NO increased HCEC proliferation and migration via time-dependent MAPK activation and eventually enhanced corneal recovery from the alkali burn.

## Introduction

The corneal epithelium is the outermost mechanical barrier of the human eye. Various corneal injuries result in corneal epithelial cell damage and break down. An injured corneal epithelium is an important source of inflammatory mediators, such as cytokines, growth factors, and proteinases; delicate cross-talk of these various signaling pathways regulates concerted wound healing events such as proliferation, migration, adhesion, and differentiation of corneal epithelial cells^1–3^. Rapid healing of the corneal epithelium and the return of an intact basement membrane can restore the eye’s normal mechanical barriers and prevent various epithelium-derived growth factors (transforming growth factor ß [TGF- ß] and platelet-derived growth factor) from leaking into the stroma. In doing so, abnormal collagen deposition and the development of corneal stromal opacity can be reduced^2^.

Nitric oxide (NO) is a small signaling molecule (free radical) with various biological functions. It is known to be an important physiological regulator of cellular proliferation^4–6^. Previous studies have reported that relatively lower concentrations of NO stimulate cellular proliferation, whereas higher concentrations of NO generally induce cell cycle arrest and growth inhibition^7^.

The specific role of NO in the wound healing process has previously been shown^8, 9^. In a skin wound model, NO is produced by various cells involved in wound healing. These include platelets, macrophages, fibroblasts, endothelial cells, and keratinocytes^9, 10^. Previously, NO deficiency resulted in impaired skin wound healing, as demonstrated in the nitric oxide synthase (NOS) knock-out mouse^11, 12^. Additionally, topical exogenous NO delivery has been shown to significantly enhance cutaneous wound healing and skin reepithelization, as well as enhance collagen deposition^13–15^. Finally, NO also acts as a vasodilator and chemoattractant for monocytes, neutrophils, and various cytokines such as IL-1 and TGF- ß, during the acute phase of the wound healing process^8, 9^.

In general, tissue injury results in the excessive accumulation of reactive oxygen species (ROS), such as superoxide and hydroxyl radical (O_2_^−^ and OH·), at the injury site^9^. These ROS play an important role in initiating the wound healing response, regulating inflammation and defending the host from invasive microorganisms. However, unregulated and excessive ROS can further damage host cells during the wound healing response. As one of the most effective antioxidants—known as a scavenging superoxide—NO converts excessive oxidative stress into less potent nitrosative stress^9^.

The role of NO in corneal wound healing has been studied for decades^16–19^. It has been shown to promote corneal epithelial wound healing in cell and animal models. However, the exact mechanism by which NO facilitates corneal wound healing remains unclear.

Therefore, in this study we investigated the effect of exogenous NO on primary culture of human corneal epithelial cells (HCEC) and in a murine corneal alkali burn model. Different concentrations of NO donor (NaNO_2_) were applied in the culture media and the cellular viability, ROS generation, autophagy, and cell proliferation pathways (mTOR and MAPKs) were evaluated. A murine corneal alkali burn model was then treated with topical NO application and the rate of corneal healing via inflammatory cytokine modulation was investigated.

## Results

### Effect of NO on HCEC viability

**Figure 1:**
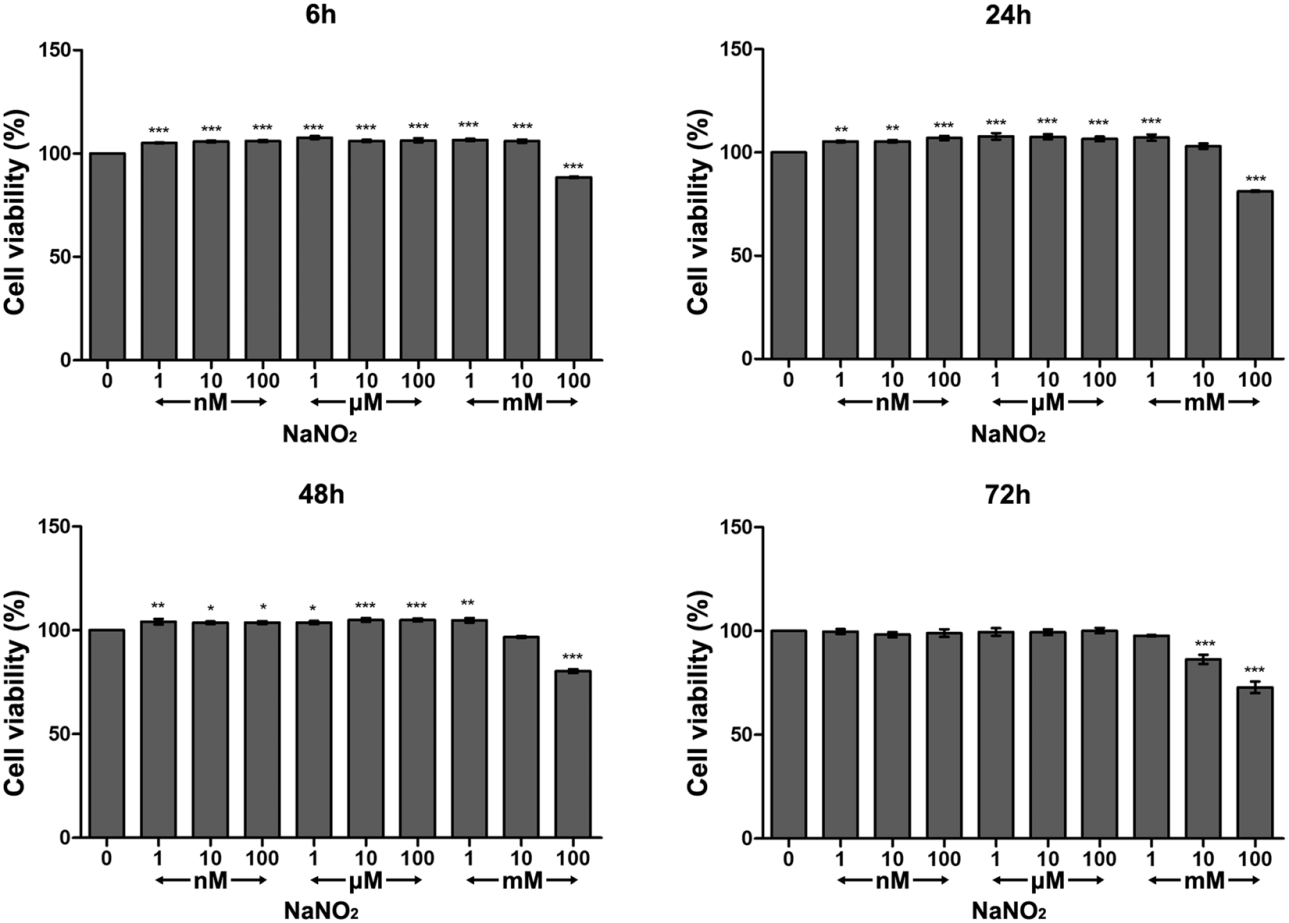
Effect of NO on HCEC viability. Cellular viability of HCECs after 6, 24, 48, and 72 h of exposure to different concentrations of NaNO_2_. A mild increase of viability was observed with low concentrations of NaNO_2_ (~1 mM) up to 48 h. A higher concentration of NaNO_2_ (100 mM) resulted in decreased viability from 6 to 72 h. Triplicates of each treatment group were used in each independent experiment. Values are presented as the mean ± SEM from four independent experiments.

The effect of NO on HCEC viability was not significant when measured at 3 h after NO application. However, relatively low concentrations of NO (1 nM to 1 mM of NaNO_2_) were seen to significantly increase HCEC viability 6 h after application, and the effect continued for 48 h. An intermediate concentration of NO (10 mM of NaNO_2_) increased HCEC viability at 6 h of exposure. However, longer exposure, up to 48 h, did not result in this effect. It is noteworthy that a high concentration of NO (100 mM of NaNO_2_) resulted in a cytotoxic effect, even at 6 h incubation, and this toxic effect only increased with the length of incubation (Fig. 1).

### Effect of NO on HCEC ROS generation

**Figure 2:**
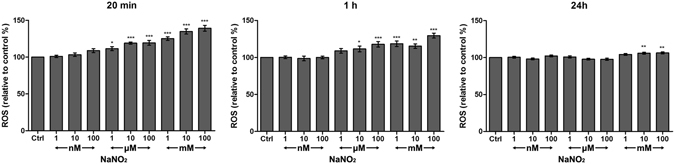
Induced ROS in HCECs following treatment with different concentrations of NaNO_2_. We found that ROS increased dose-dependently with the addition of NO. The effect was most prominent at 20 min after addition of NaNO_2_. Values are presented as means ± SEM (n = 3) and are calculated as a percentage of negative control (0 μg/mL treated groups); P values were calculated compared to negative control. *p < 0.05, **p < 0.01, ***p < 0.001.

As a relatively unstable gas, NO only lasts for several seconds. Therefore, we measured intracellular ROS change at 20 min, 1 h and 24 h after NO application. We found that NO induced dose-dependent increases of intracellular ROS in HCEC at 20 min and 1 h. Exposure to low concentrations of NO (equal or less than 100 nM of NaNO_2_) had no significant effect on ROS generation. When measured at 24 h after exposure, a mild but significant elevation of ROS in HCEC persisted at the 10 mM and 100 mM concentrations of NaNO_2_ exposure (Fig. 2).

### Effect of NO on autophagy and mTOR pathway of HCEC

**Figure 3:**
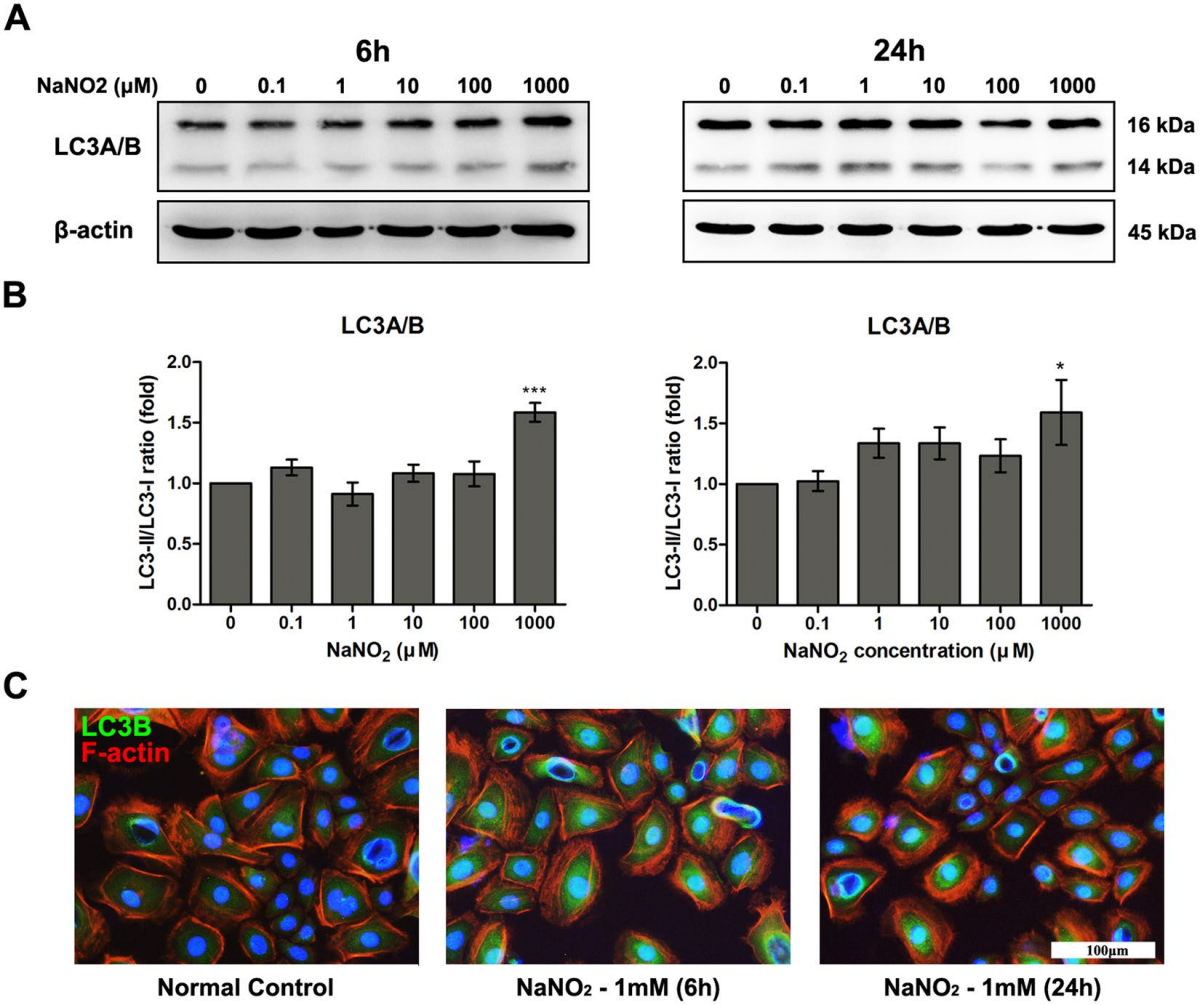
Effect of NO on autophagy of HCEC. The expression levels of autophagy signal, LC3A/B proteins were measured by western blot analysis (**A**). The inactive I form is 16 kDa and the active II form is 14 kDa. Relative densitometry (**B**) was calculated as a percentage of the control, and all values (mean ± SEM) were obtained from three independent experiments; each independent experiment was performed in triplicate (*p < 0.05, ***p < 0.001). The increased LC3B expression (shown in green) resulting from high a concentration (1 mM) of NaNO_2_ was demonstrated with immunocytochemical assay after 6 and 24 h incubation (**C**). Full size images of blots are available in the Supplementary information.

**Figure 4:**
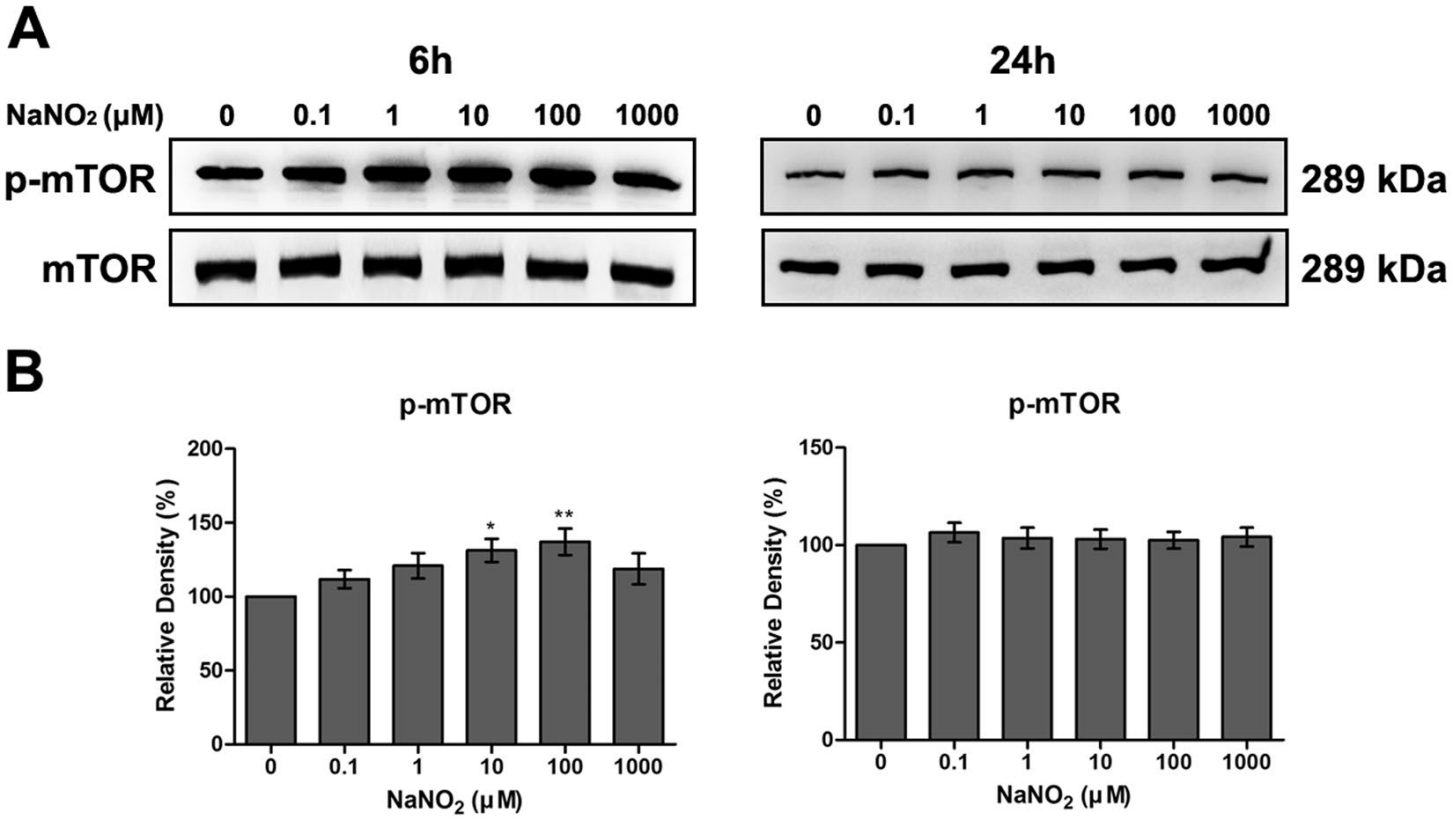
Effect of NO on mTOR pathway of HCEC. Expression levels of phosphorylated mTOR (p-mTOR) and mTOR, detected by western blot analysis (**A**) and relative densitometry (**B**), were calculated as a percentage of the control. Increased phosphorylation of mTOR was observed at 6 h exposure to 10 μM and 100 μM of NaNO_2_. Full size images of blots are available in the Supplementary information. Values are presented as mean ± SEM and were obtained from three independent experiments; each independent experiment was performed in triplicate. *p < 0.05, **p < 0.01.

We investigated the effect of NO on the cellular autophagy system using the signal alteration of LC3A/B, the autophagy marker (Fig. 3). Considering the short half-life of NO, we collected protein from HCECs at 6 and 24 h after exposure to NO. With the activation of autophagy, LC3A/B II form increases relative to LC3A/B I form. Exposure to a higher concentration (1 mM) of NaNO_2_ for 6 and 24 h significantly activated autophagy pathway in HCECs. A slightly increased LC3A/B II form was observed with exposure to 1 to 100 μM of NaNO_2_ with 24 h incubation, but was statistically insignificant. Additionally, NO exposure appeared to slightly increase phosphorylated mTOR (p-mTOR) at 6 h, especially at the 10 and 100 μM concentrations. However, the activation of mTOR showed no significant change with NO exposure at 24 h (Fig. 4).

### *In vitro* scratch assay and HCEC migration assay

**Figure 5:**
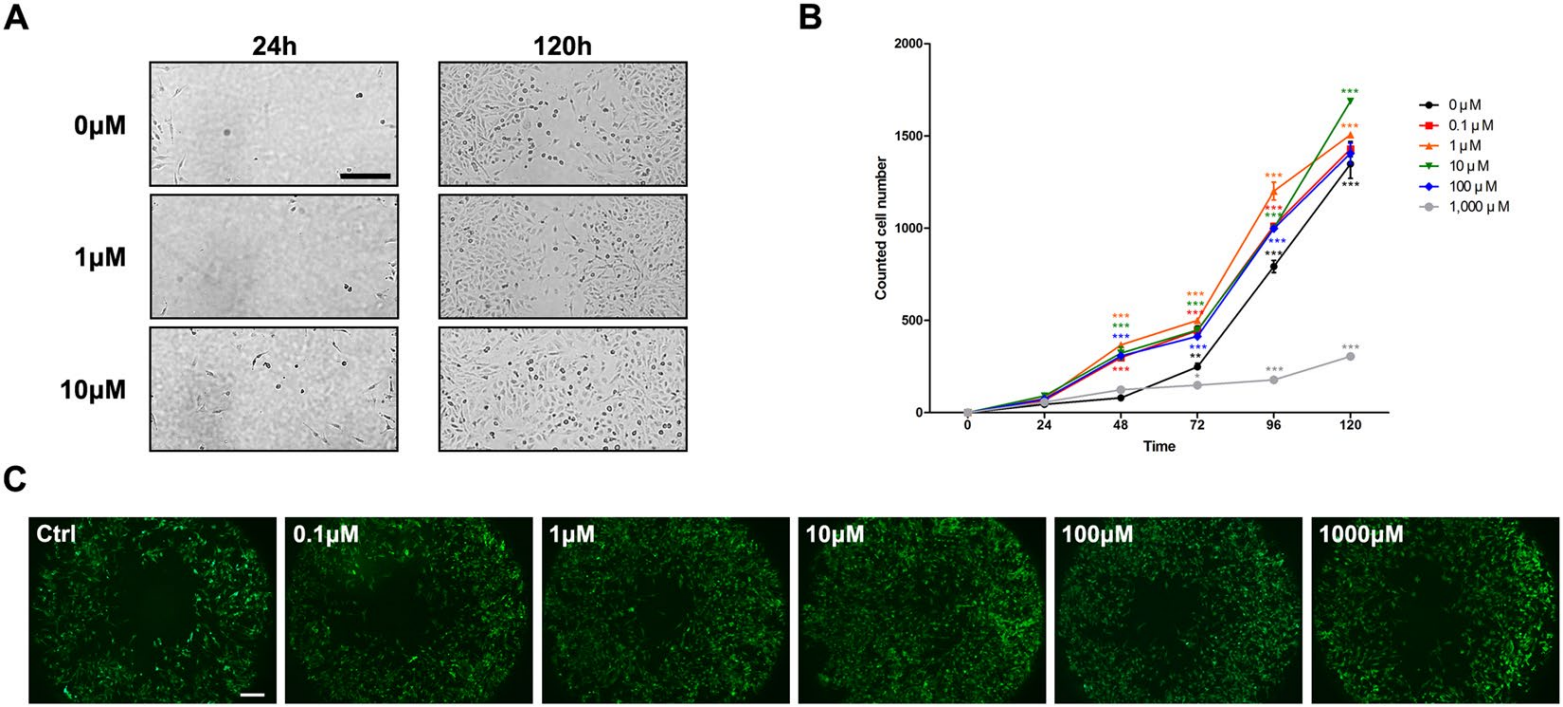
*In vitro* scratch assay and HCEC migration assay. (**A**) In the scratched area, HCEC repopulation was observed up to 120 h. The addition of NaNO_2_ increased HCEC repopulation significantly. The scratched area was completely covered at 120 h following the addition of 10 μM NaNO_2_ compared to the negative control (0 μM). (**B**) The number of cells counted at the scratched area is shown. Cell numbers were significantly increased with the addition of NaNO_2_ (0.1 to 100 μM); NaNO_2_ (10 μM) showed the highest cell concentration when measured at 120 h. However, a high concentration of NaNO_2_ (1000 μM) significantly decreased cell counts in the scratched area compared to the negative control. (**C**) An image of the central detection zone in cell migration assay was captured, in order to compare the pattern of cell migration. The positive effect of NaNO_2_ on HCEC migration was most prominent in conjunction with the 10 μM concentration. *p < 0.05, **p < 0.01, ***p < 0.001.

The scratch assay confirmed that NO significantly increased HCEC migration into the cell- free area, as shown in Fig. 5. The most prominent acceleration of HCEC migration was observed with the addition of 10 μM of NaNO_2_. However, a high concentration of NaNO_2_ (1 mM) induced significant cytotoxicity and resulted in decreased HCEC migration.

### Effect of NO on MAPK activation of HCEC

**Figure 6:**
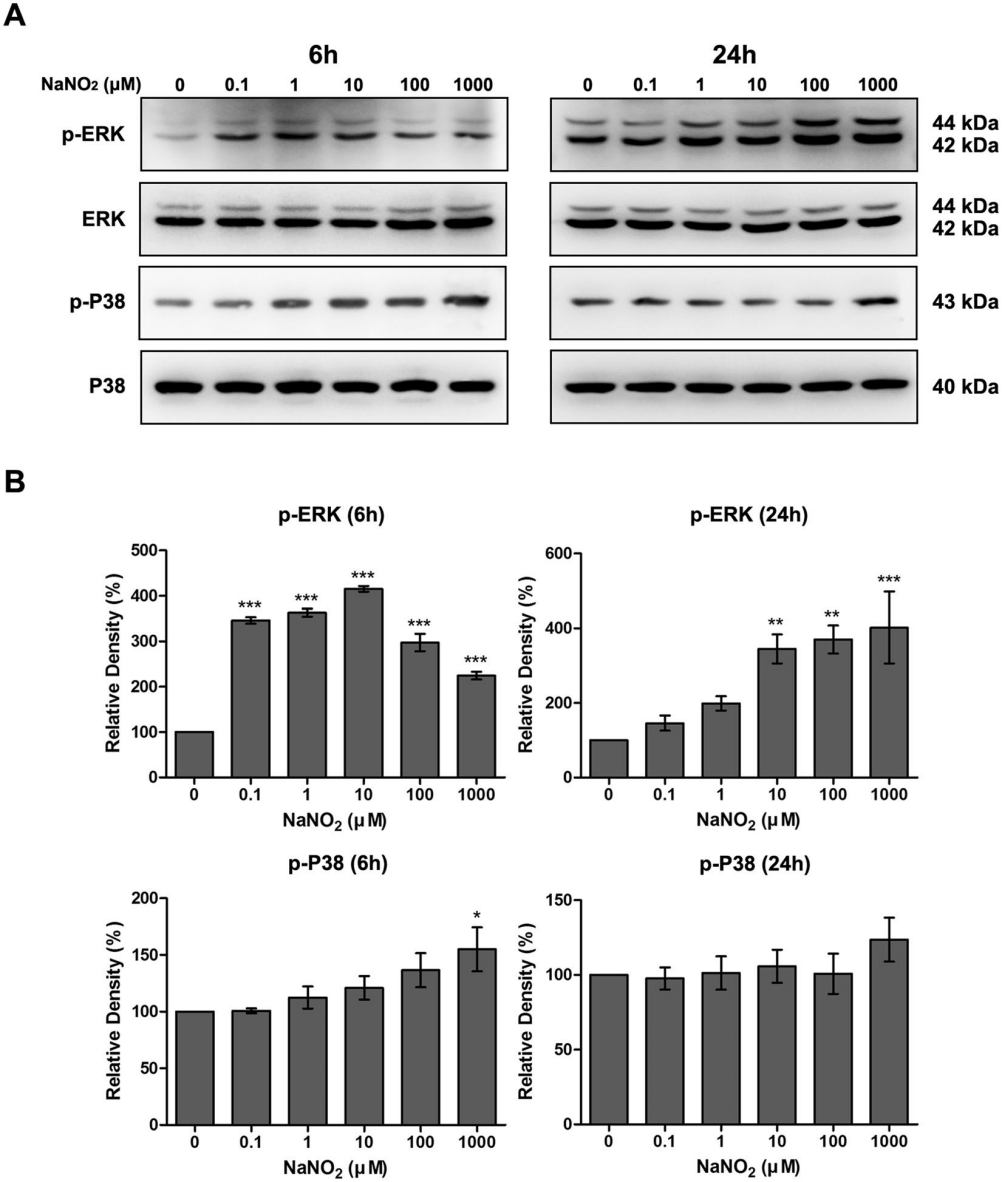
Effect of NO on MAPK activation of HCECs. The expression levels of phosphorylated ERK (p-ERK) and phosphorylated p38 MAPK (p-p38) were detected by western blot analysis after 6 and 24 h exposure to various concentrations of NaNO_2_ (**A**). Relative densitometry (**B**) was calculated as a percentage of the control. It is clear that ERK phosphorylation increased at 6 and 24 hr. However, p38 MAPK phosphorylation increased only at 6 h, and with a high concentration (1000 μM). Full size images of blots are available in the Supplementary information. Values are presented as mean ± SEM and were obtained from three independent experiments; each independent experiment was performed in triplicate. *p < 0.05, **p < 0.01, ***p < 0.001.

Our study also found that MAPK phosphorylation was modulated by NaNO_2_; both time- and dose-dependently, NaNO_2_ (0.1 to 1000 μM) significantly increased ERK phosphorylation of HCECs when measured at 6 h after exposure. However, when measured at 24 h after exposure, significantly enhanced ERK phosphorylation was only detectable in the presence of relatively high concentrations (10 to 1000 μM) of NaNO_2_. Furthermore, p38 MAPK phosphorylation showed increasing tendency after 6 h exposure to various concentrations of NaNO_2_. However, statistical significance was only achieved with the 1000 μM concentration. After 24 h exposure, p38 MAPK phosphorylation was normalized (Fig. 6).

### *In vivo* effect of NO on corneal wound healing

**Figure 7:**
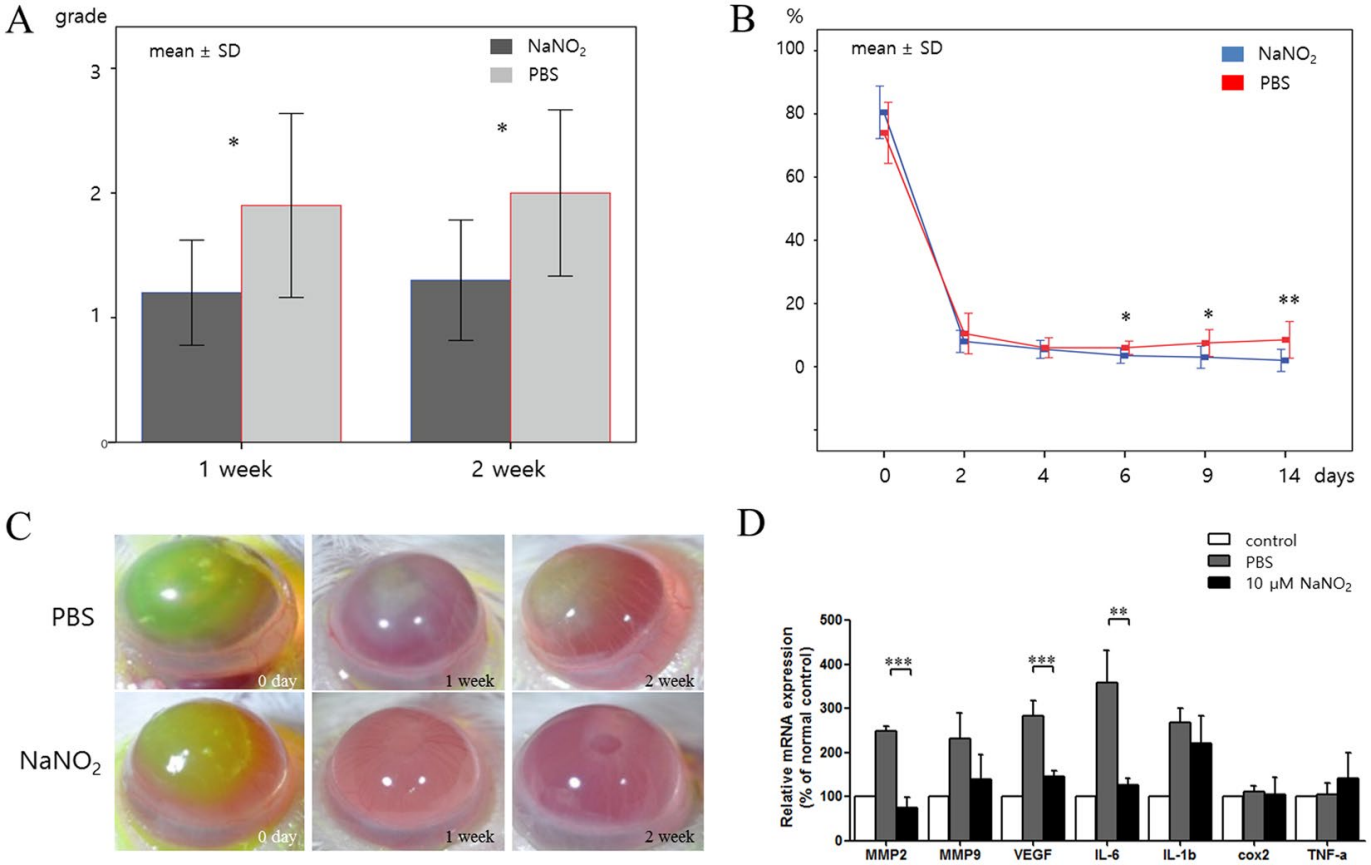
*In vivo* effect of NO on corneal wound healing following alkali burn. (**A**) Corneal opacity of NO-treated corneas was significantly alleviated than those in the PBS control group, both at 1 and 2 weeks. Higher grade means higher opacity. (**B**) Corneal epithelial defect size (%) was traced. Corneal epithelial healing was faster in NO-treated corneas and the defect size was smaller compared to those in the PBS control group, from days 6 to 14. (**C**) Representative photographs of the alkali-burned murine cornea at 0, 7, and 14 days after injury. The green area represents the corneal epithelial defect stained with fluorescein. Corneal opacity is prominent in PBS-treated cornea compared to the NaNO_2_-treated cornea. Pupil is clearly visible in NaNO_2_-treated cornea at 2weeks with no epithelial defect. (**D**) Inflammatory cytokine expression from cornea measured at 2 weeks after injury revealed significantly decreased mRNA expression of MMP2, VEGF, and IL-6 in NaNO_2_-treated corneas. **p < 0.01, ***p < 0.001

Corneal wound healing promotion by NO was further verified in a mouse model. The concentration 10 μM concentration of NaNO2 was selected based on both its cell viability/migration enhancing effect and its prominent ERK phosphorylation effect (Fig. 6). Topical treatment with 10 μM of NaNO_2_ solution for 1 week after corneal alkali burn significantly enhanced corneal epithelial healing and decreased corneal opacity induced by chemical burn (Fig. 7A–C). Furthermore, the expression of key inflammatory cytokines was measured 2 weeks after the injury. It revealed that NO treatment significantly decreased the expression of MMP2, VEGF, and IL 6 in the injured corneas compared to the PBS-treated control (Fig. 7D).

## Discussion

In this study, we found that NO had a dose-dependent bimodal effect on corneal wound healing. Namely, low concentrations of NaNO_2_ significantly enhanced HCEC viability and promoted cornea wound healing, whereas higher concentrations of NaNO_2_ increased HCEC cytotoxicity. In addition, the wound healing effect of NO is related to the time-dependent activation of MAPK (ERK and p38 kinase) pathways in HCECs.

The dose-dependent effect of NO on cellular physiology has been reported previously. Lower concentrations (picomolar or nanomolar) of NO have been seen to exert a direct and positive effect on cellular proliferation, whereas higher concentrations (milimolar) have indirect cytotoxic effects through both oxidation and nitrosative stresses^6, 20^. Furthermore, NO at low concentrations (2 to 50 µM) has protected bone marrow stromal and embryonic stem cells from apoptosis and instead promoted proliferation^21–23^. It has also prevented cultured corneal fibroblasts from serum deprivation-induced apoptosis in a dose-dependent manner^18^. However, at higher concentrations, NO interacts with superoxide and can produce peroxynitrite (ONOO-), which induces apoptosis with caspase activation and mitochondrial damage^24^. Nucleic acid and proteins can be damaged by nitrosylation or nitration of amines, thiols, and tyrosine residues in the presence of high concentrations of NO^25^. Peroxynitrite can also damage corneal endothelial cells and induce corneal edema^26^.

The cellular proliferative and survival effects of NO are still poorly understood. However, the regulation of MAPK activity by NO has been suggested as one of the potential mechanisms underlying NO’s impact on cell proliferation. It is also known that NO can affect the cell cycle via both cyclic-guanosine monophosphate (cGMP)- dependent and cGMP- independent mechanisms. These two mechanisms can influence epidermal growth factor receptor (EGFR)/RAS/MAPK (ERK) pathway activation^6^. As has previously been known, MAPK pathways regulate diverse cellular activities including mitosis, mobility, survival, apoptosis, and differentiation. Additionally, these pathways can be activated by various external stimuli, including growth factors, ultraviolet light, and many types of chemicals^27, 28^. In particular, ERK is known to be preferentially activated by growth factors, whereas the activation of c-Jun N-terminal kinase (JNK) and p38 MAPK is largely dependent on various stress-inducing stimuli such as inflammatory cytokines^27, 28^. In one report, a low concentration of NO (10 µM) increased neural stem cell proliferation via activation of p21RAS and the MAPK pathway^29^.

Corneal epithelial wound healing is a complex process, and HCEC proliferation, migration, and differentiation are delicately coordinated by many cytokines^3^. It has previously been reported that activation of the ERK pathway is important in HCEC proliferation, whereas the activation of p38 MAPK is more related to HCEC migration^30^. For example, hepatocyte growth factor (HGF) can activate both ERK and p38 MAPK, and their coordination is essential for HCEC proliferation and migration, ultimately leading to the healing of corneal epithelial wounds^30^. The ERK pathway is one of the primary pathways involved in epidermal growth factor (EGF) and nerve growth factor (NGF)- induced HCEC proliferation and migration^31, 32^. For example, impaired ERK phosphorylation in diabetic rats resulted in delayed corneal epithelial wound healing^33^. Our finding that the activation of ERK and p38 MAPK were increased in a time-dependent manner by NO is consistent with those of previous studies. We observed that the activation of p38 MAPK occurs early, at 6 h after NO treatment, whereas the activation of ERK was increased both at 6 and 24 h after NO treatment. During the healing process, cell migration from adjacent HCECs is important in order to initially cover the wounded area. We hypothesize that the early activation of p38 MAPK may indicate increased cell migration, and that the activation of ERK may signal an increased supply of new HCECs by way of active proliferation. It is noteworthy that ERK activation can also induce endogenous NO production and facilitate fibroblast proliferation and migration^34^. Therefore, both NO and ERK activation can construct a positive feedback loop and enhanced cellular proliferation.

Another interesting finding of our study is that co-culturing HCECs with NO can mildly increase cellular ROS in a dose-dependent manner for up to 1 h. As a key molecule representing reactive nitrogen species, NO can interact with an ROS, such as superoxide, by formation of peroxynitrite (ONOO-). Therefore, reactive oxygen and nitrogen species are generated together during various cellular stresses^9^. We believe that in our study, exogenous NO added in the culture media directly increased intracellular ROS in corneal cells. However, ROS can modify many intracellular signaling pathways, including protein phosphatases, protein kinases and transcription factors^35^. While a high concentration of ROS provides a direct effector mechanism for cellular death, a mild-to-moderate oxidative stress with low ROS concentration can enhance the proliferation of hepatic cells by activation of MAPKs^36–38^. Recently, it was reported that the proliferative effect of EGF on HCECs is mediated by ROS, both in physiologic and diseased conditions^31^.

The cytotoxicity observed in high concentrations (10 mM and 100 mM) of NaNO_2_ can be attributed to osmotic stress due to increased Na concentration in the medium^39^. The addition of 10 mM and 100 mM NaNO_2_ may cause osmolarity to increase in the culture media by 20mOsM and 200mOsM, respectively, while the addition of less than 1 mM NaNO_2_ has little effect on osmolarity (less than 2mOsM). Therefore, sodium chloride (NaCl) was used as an osmotic agent to test the effect of osmotic stress on HCEC viability. Adding 10 mM and 100 mM NaCl in the culture media significantly increased HCEC cytotoxicity and ROS generation (Supplementary Figures 1 and 2).

In this study, we found that NO can modulate inflammatory cytokine expression on the ocular surface and enhance corneal wound healing. In a previous report, it was discovered that NO could attenuate acidic pH induced cyclooxygenase 2 expression in corneal endothelial cells^40^. Inflammatory cytokines are essential to initiate the early phases of wound healing^3, 41^. However, prolonged expression of inflammatory cytokines can result in chronic persistent epithelial defects and corneal opacity^3^. Therefore, control of inflammatory cytokines is essential for optimal corneal wound healing. We found that NO could decrease the expression of MMP2, VEGF, and IL-6 in alkali- burned cornea significantly. This finding suggests that inflammatory processes may possibly be successfully controlled in NO treated corneas (Supplementary Figure 3).

Another positive aspect of NO in corneal wound healing is the protection of the injured cornea from bacterial infections during re-epithelialization. NO plays an important role in the innate immune response to bacterial pathogens. The mouse corneal epithelium infected with *P. aeruginosa* did not produce NO during the infection^19^. Therefore, an exogenous supply of NO has the potential to promote bacterial clearance and corneal wound healing.

In summary, the current study revealed that low concentrations of NO (0.1 to 10 μM NaNO_2_) could increase HCEC proliferation and migration capacity. The activation of both ERK and p38 MAPK pathways was involved in this process. In addition, topical treatment of NO enhanced corneal wound healing after alkali burn *in vivo*. These results may be used for future development of NO-based therapy for ocular surface diseases.

## Materials and Methods

### Cell cultures

In this study, HCECs were purchased from American Type Culture Collection (ATCC, Rockville, MD, USA). Cells were resuspended in corneal epithelial cell basal medium (ATCC) supplemented with a growth kit (ATCC). The cells were plated in 75 cm^2^ tissue flasks, and then maintained at 37 °C in a 5% CO_2_ and a 95% humidified atmosphere. Culture medium was changed every 3 d and the cells were passed using 0.05% Trypsin-EDTA (Gibco BRL, CA, USA). A passage number of ≤5 was used in this study.

### Cell viability assay

We assessed cell viability using CCK-8 reagent (Dojindo Molecular Technologies, Inc., Kumamoto, Japan) according to the manufacturer’s protocol. Briefly, HCECs were cultured at 4 × 10^3^ cells/well in a 96-well plate and incubated for 24 h. Following the adherence of cells, sodium nitrite (NaNO_2_; Sigma_-_Aldrich, St. Louis, MO, USA) was applied to cells for 3 h, 6 h, 24 h, and 48 h, dose-dependently: 0, 0.001, 0.01, 0.1, 1, 10, 100, 1000, 10000, 100000 μM. Following the appropriate period of incubation, 10% (v/v) of the CCK-8 solution in culture media was prepared and the absorbance at 450 nm was measured after 2 h incubation of HCEC with reagent.

### Measurement of reactive oxygen species (ROS)

The generation of ROS was detected using a fluorometric intracellular ROS kit (Sigma-Aldrich); HCECs were seeded on 96 black well plates (4 × 10^3^ cells/well) 24 h before the treatment. Next, NaNO_2_ was applied (at concentrations of 0, 0.001, 0.01, 0.1, 1, 10, 100, 1000, 10000, 100000 μM) in HCEC for 20 min, 1 h, and 24 h. The mixture of ROS detection reagent stock solution and assay buffer was then treated for 30 min and the fluorescence intensity was measured (λ_ex_ = 540/λ_em_ = 570 nm). In our study, 20 uM of hydrogen peroxide was used for the standard curve by serial dilution and 50 uL of appropriate samples, cells, or supernatants were transferred into 96 black well plates. Then, 50 uL of catalyst and 100 uL of DCFH solution was added and incubated at room temperature for 30 min. Finally, fluorescence was read at 480 nm excitation/530 nm emission.

### *In vitro* scratch assay

*In vitro* corneal epithelial wound healing modelling was performed using a scratch assay. Confluent monolayers of HCECs were linearly scratched with a comb to create cell-free areas. Various concentrations of NaNO2 were then added to the culture media and HCEC migration into the scratched areas was monitored daily for 5 d. No addition of NaNO_2_ was used as a negative control. Cell numbers in the initially-scratched areas of each picture were counted using Image J software (http://imagej.nih.gov/ij/).

### HCEC migration assay

The effect of NO on cellular migration rates was assayed using the Cell Migration Assay, TC (Enzo Life Science, Farmingdale, NY, USA). First, HCECs were seeded onto the 96-well plate at 4 × 10^3^ cells/well and maintained at 37 °C in 5% CO_2_ for 24 h. After checking the adherence of cells, the stopper, which was located at the center of each well, was removed to create a detection zone for HCEC migration. Then, different concentrations of NaNO_2_ (0.1 μM, 1 μM, 10 μM, and 100 μM) were added to the culture media and the migration of HCECs to the detection zone was investigated in a time-dependent manner (24 h, 48 h, 72 h, 96 h and 120 h).

### Western blot analysis

In this study, we investigated the effect of NO on modulation of mTOR, autophagy, and MAPKs pathways after 6 and 24 h of exposure to NO. These two time points were selected due to the short half-life of NO. HCECs were lysed in ice-cold RIPA buffer (50 mM Tris-HCl (pH 8.0), 150 mM NaCl, 1% NP-40, 0.5% deoxycholate, and 0.1% SDS) for 30 min. The debris was removed by centrifugation at 16,000 g for 1 min. Equal amounts (20 μg) of total cell protein were separated by SDS-polyacrylamide gel electrophoresis (SDS-PAGE) and transferred to a PVDF membrane. After blocking with 5% BSA in TTBS buffer (10 mM Tris, pH 8.0, 150 mM NaCl, 0.1% Tween 20) for 1 h at room temperature, membranes were incubated overnight at 4 °C with the following primary antibodies: rabbit anti-LC3A/B (1:1000; catalog number: 12741; Cell Signaling, Beverly, MA, USA), rabbit anti-phospho-mTOR (1:1000; catalog number: 5536; Cell Signaling), rabbit anti-mTOR (1:1000; catalog number: 2983; Cell Signaling), rabbit anti-phospho ERK1/2 (T202/Y204) (1:1,000; catalog number: 4370), rabbit anti-ERK1/2 (1:1,000; catalog number: 4695), rabbit anti-phospho p38 (T180/Y182) (1:1,000; catalog number: 4511), rabbit anti-p38 (1:1,000; catalog number: 8690), and mouse anti-β-actin (1:10,000; catalog number: sc-47778; Santa Cruz, Biotechnology, Dallas, Texas, USA). The membranes were incubated with peroxidase-conjugated secondary antibody for 1 h at room temperature. Blots were developed using an enhanced chemiluminescence (ECL) kit (catalog number: RPN2232; GE healthcare, Buckinghamshire, UK) and visualized using a Fujifilm Image Reader LAS-3000 (Fujifilm, Tokyo, Japan). Each experiment was repeated at least three times, and the densitometric analysis was performed using the Multi Gauge V3.0 (Fujifilm Life Science, Tokyo, Japan).

### Effect of NO on *in vivo* corneal wound healing process

To investigate the *in vivo* effect of NO on corneal wound healing, Balb/c mice (20 males) were used. Animals were treated in compliance with the ARVO *Statement for the Use of Animals in Ophthalmic and Vision Research*. Experimental protocol was approved by Institutional Animal Care and Use Committee of Dongguk University Ilsan Hospital (reference number: 2016–03146). Alkali burn was induced on the right corneas of the mice. Briefly, after the application of topical anesthesia with 0.5% proparacaine hydrochloride (Alcaine®, Alcon laboratory, Fort Worth, TX, USA), a 0.1 N NaOH-soaked filter paper was applied on the central cornea of each mouse for 1 minute and the cornea washed with 1 ml of PBS. No intervention was applied to the left corneas. From the day of alkali burn, 10 mice were treated with topical applications of 10 μM NaNO_2_ mixed with PBS, at the rate of one drop every 6 h for 1 week. The other 10 control mice were treated with PBS every 6 h for 1 week. Ocular surface pictures of the right eyes were taken at 0, 2, 4, 7, and 14 d after alkali burn. Corneal epithelial defects were measured as a percentage of defect after fluorescein staining. Corneal opacity was graded as 0: no opacity; 1: mild opacity, but easy visualization of iris vessels; 2: moderate opacity with significant obscuring of iris vessel visualization; 3: severe opacity with no iris vessel visualization. At 2 weeks after alkali burn, all mice were sacrificed and both corneas were harvested for RNA extraction.

### Real time PCR for inflammatory cytokines

Total RNA was isolated from mouse corneal tissues using the Trizol (Invitrogen) method, and the purity and yield of the RNA were determined spectrophotometrically. One microgram of total RNA from each sample was reverse transcribed into cDNA using the PrimeScript^TM^ RT Master Mix (Takara Bio Inc., Otsu, Shiga, Japan) in a total volume of 20 µl according to the manufacturer’s instructions. Five hundred nanograms of cDNA were included in a template on the Light Cycler 480 (Roche, Mannheim, Germany) using the SYBR Green Premix EX Taq kit (Takara Bio). The primer sequences from this study are shown in Table 1. The dissociation curves were generated to check for the specificity of primer annealing to the template. The expression level of each target gene was calculated using the comparative threshold cycle method (2^−ΔΔCt^) with GAPDH as the control gene. All PCR assays were performed in triplicate.

**Table 1:** Primers for real time PCR.

Gene	Forward Sequence (5′-3′)	Reverse Sequence (5′-3′)
MMP-2	ATG TGT CTT CCC CTT CAC TTT C	GGT CAT CAT CGT AGT TGG TTG T
MMP-9	CTT CCC CAA AGA CCT GAA AAC	ACT GCT TCT CTC CCA TCA TCT
VEGF	ACT ATT CAG CGG ACT CAC C	AAC CAA CCT CCT CAA ACC
TNF-a	CGA GTG ACA AGC CTG TAG CCC	GTC TTT GAG ATC CAT GCC GTT G
IL-1β	ACT CCT TAG TCC TCG GCC A	TGG TTT CTT GTG ACC CTG AGC
IL-6	TGG AGT CAC AGA AGG AGT GGC TAA G	TCT GAC CAC AGT GAG GAA TGT CCA C
Cox2	GTC TTT GGT CTG GTG CCT GG	GCT CAT CAC CCC ATT CAG GA
GAPDH	CTA CCC CCA ATG TGT CCG TC	GCT GTT GAA GTC GCA GGA GAC

### Statistical analysis

Data are presented as mean ± standard error, and statistical significance was determined using a one-way ANOVA, followed by the Bonferroni multiple comparison test. In this study, *P* < 0.05 was regarded as significant and calculations were completed with GraphPad Prism Ver. 5.01 (GraphPad Software, Inc., La Jolla, CA, USA).

### Data availability statement

All data generated or analysed during this study are included in this published article (and its Supplementary Information files).

## Electronic supplementary material

Supplementary Information
