# Supplementary material for: Effect of Nitric Oxide on Human Corneal Epithelial Cell Viability and Corneal Wound Healing
Source: Sci Rep. 2017 Aug 14;7:8093. doi: 10.1038/s41598-017-08576-9 (PMC5556055; doi:10.1038/s41598-017-08576-9)

Effect of Nitric Oxide on Human Corneal Epithelial Cell Viability and Corneal Wound Healing

Joo-Hee Park PhD[a], Ja-Yeon Kim MS[a], Dong Ju Kim MD [a], Martha Kim MD [a], Minwook Chang MD PhD[a], Roy S. Chuck MD, PhD[b], Choul Yong Park MD, PhD[a]*

Supplementary Information

Full length gel of electrophoresis

Figure 3.

6h-LC3A/B

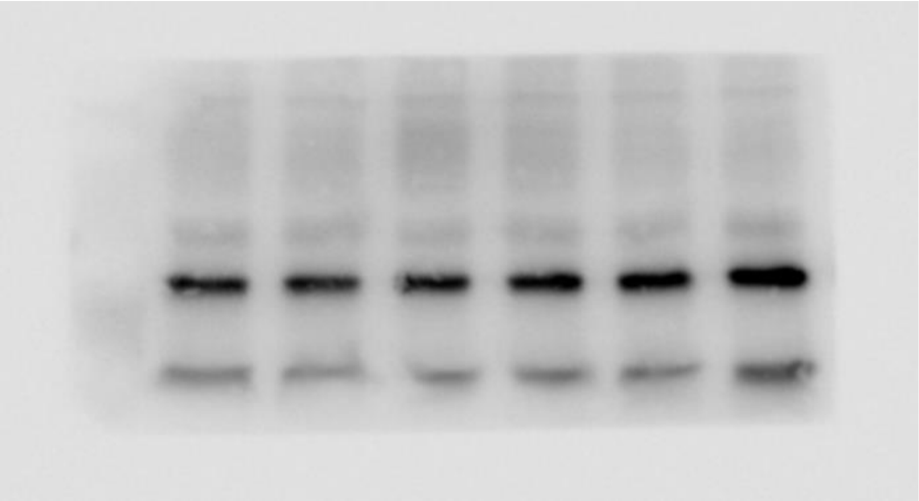

24h-LC3A/B

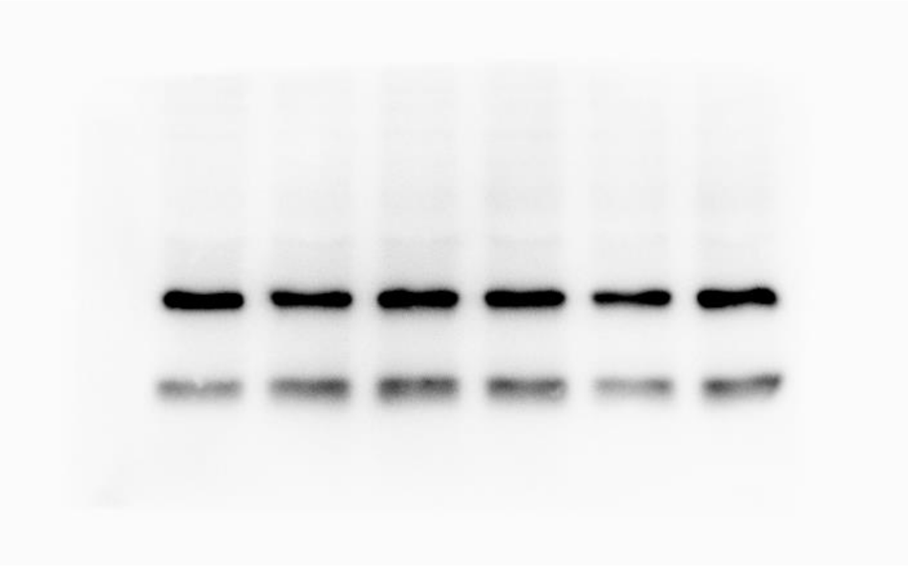

6h-actin

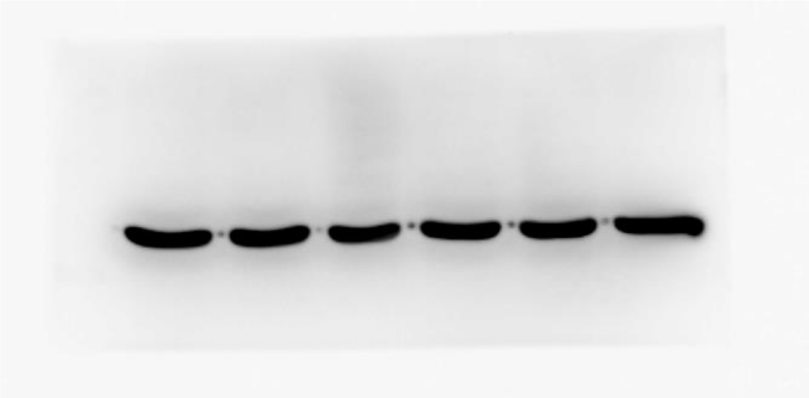

Figure 4.

6h-pmTOR

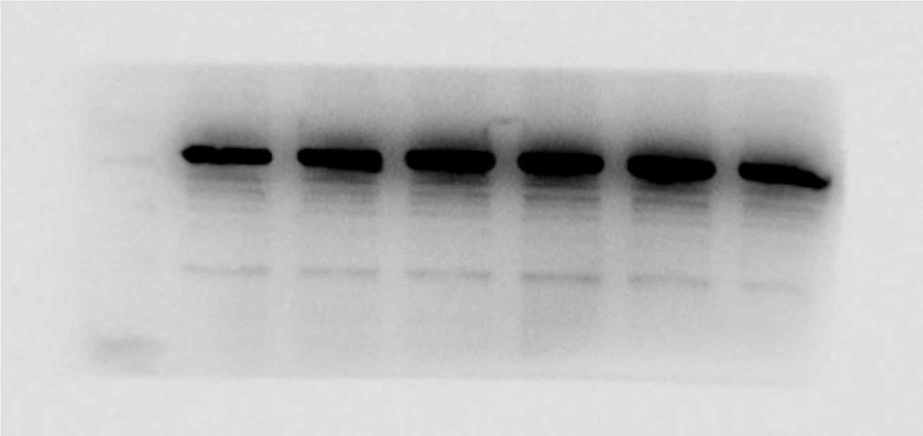

24h-pmTOR

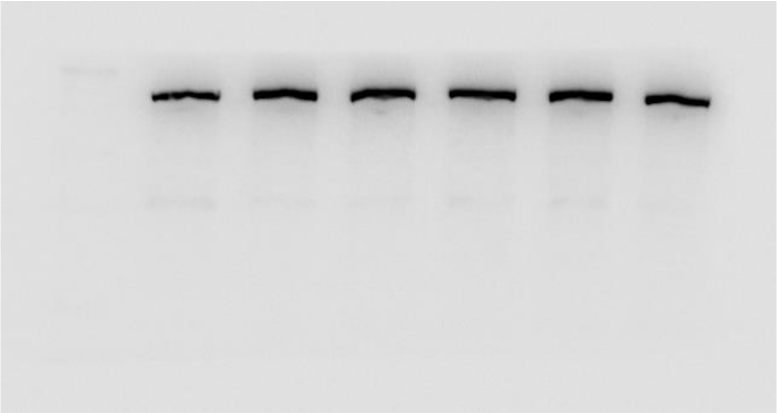

24h-mTOR

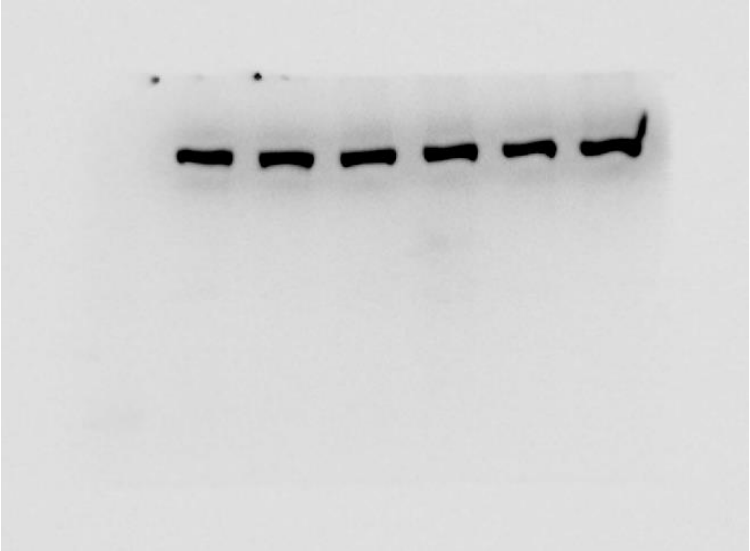

24h-actin

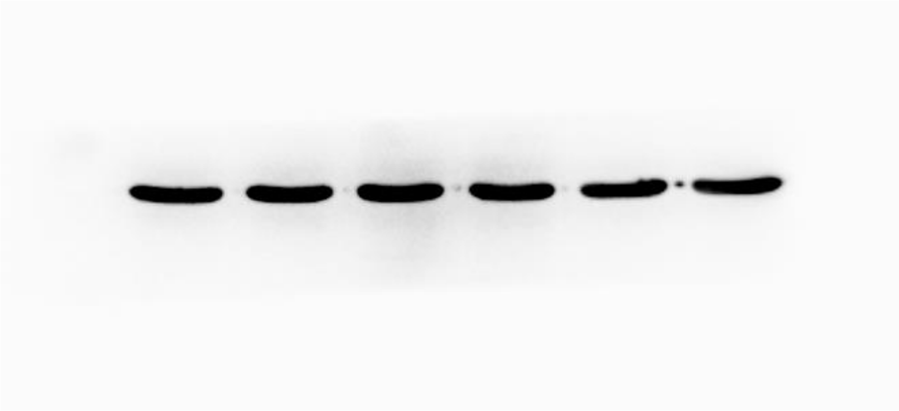

Figure 6.

6h-pERK

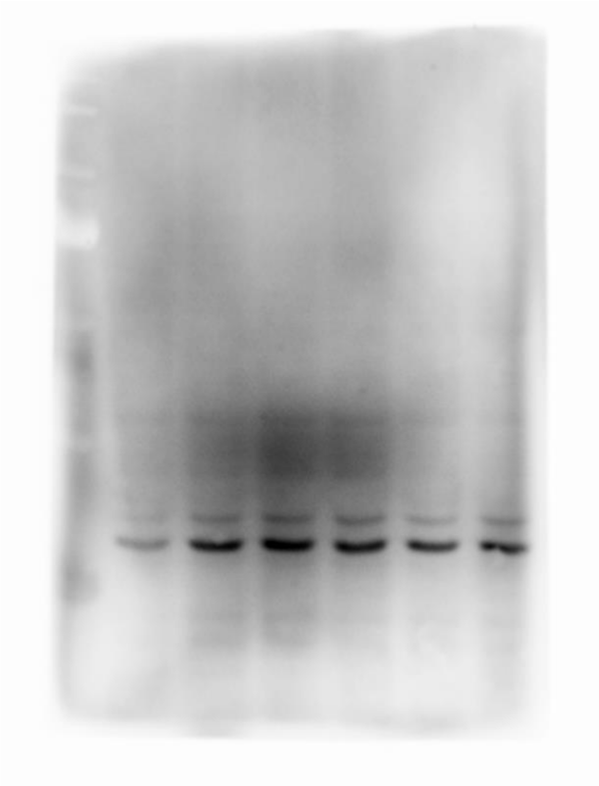

6h-ERK

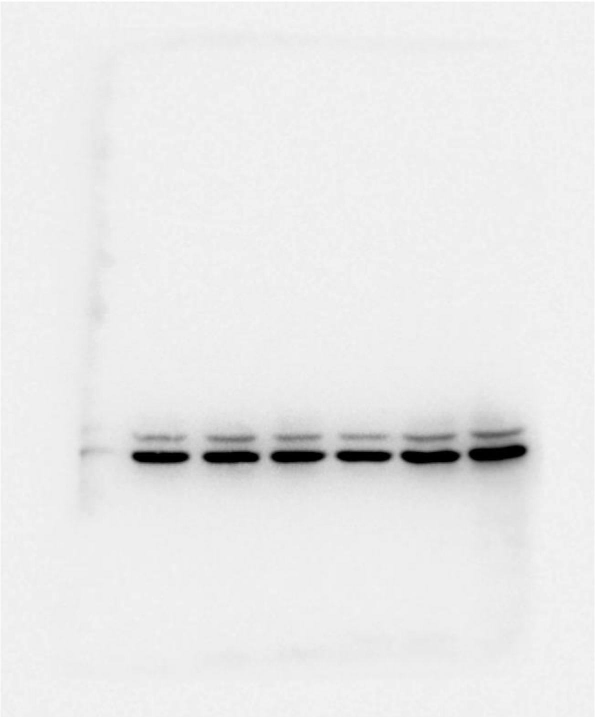

6h-pP38

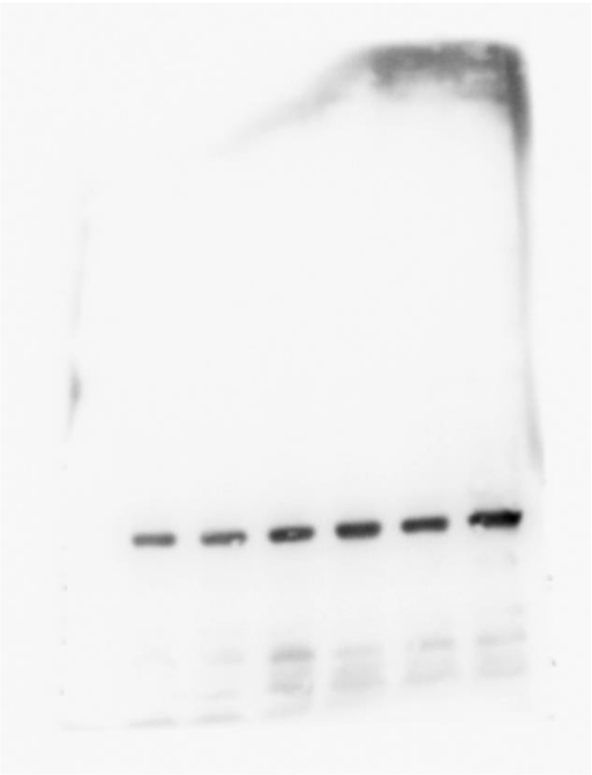

6h-P38

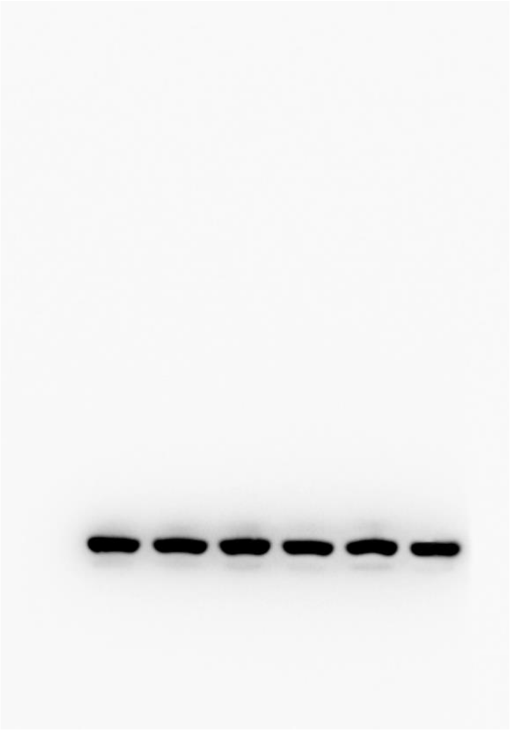

24h-pERK

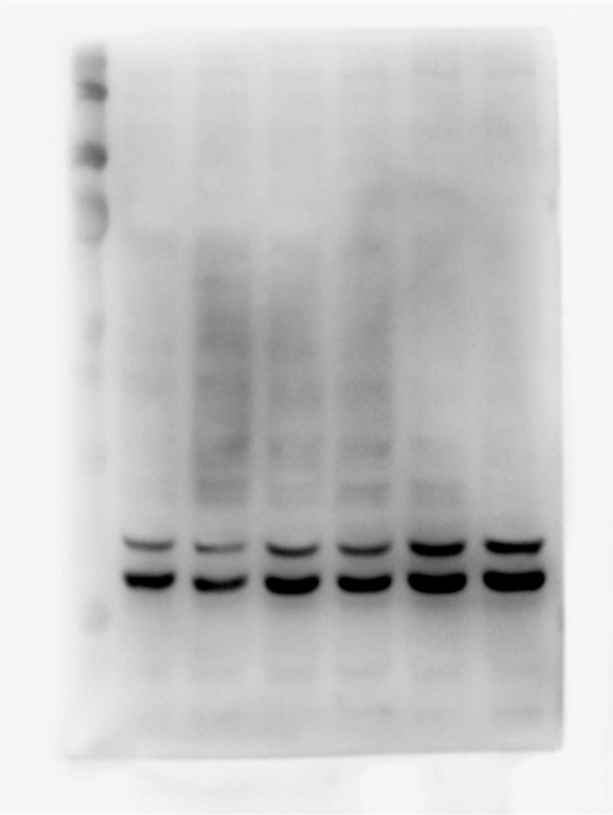

24h-ERK

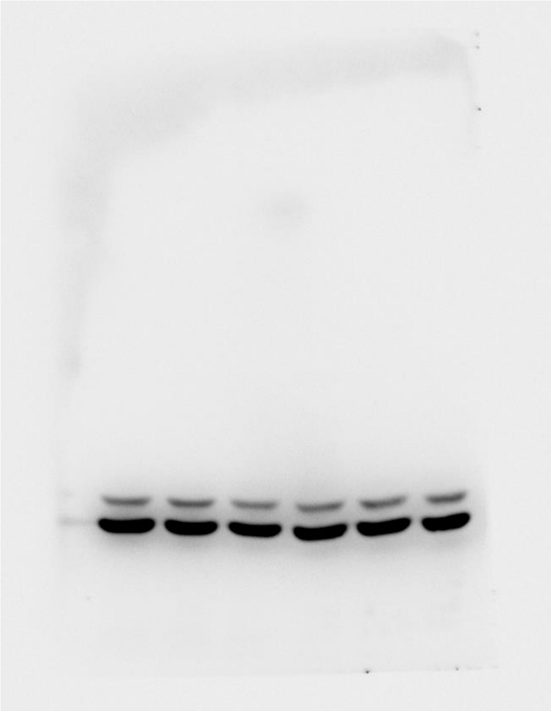

24h-pP38

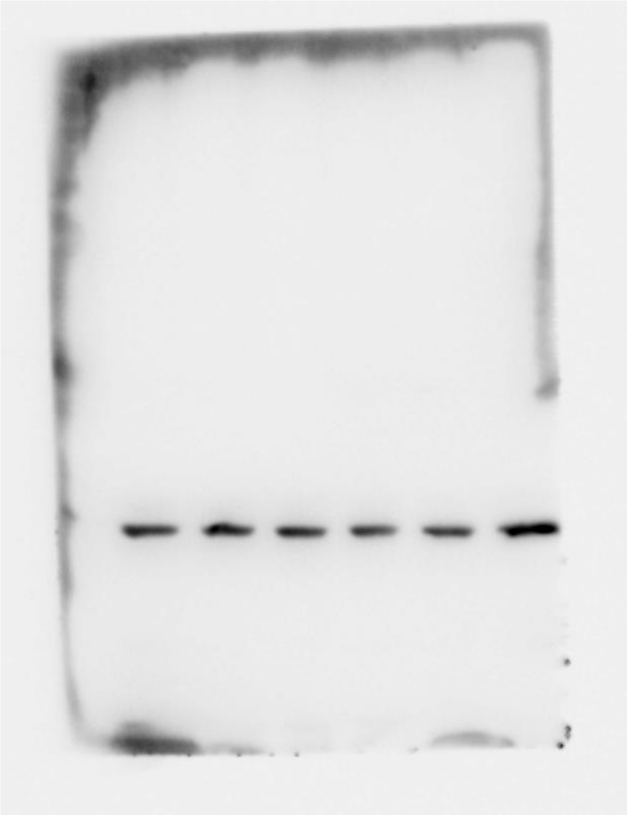

24h-P38

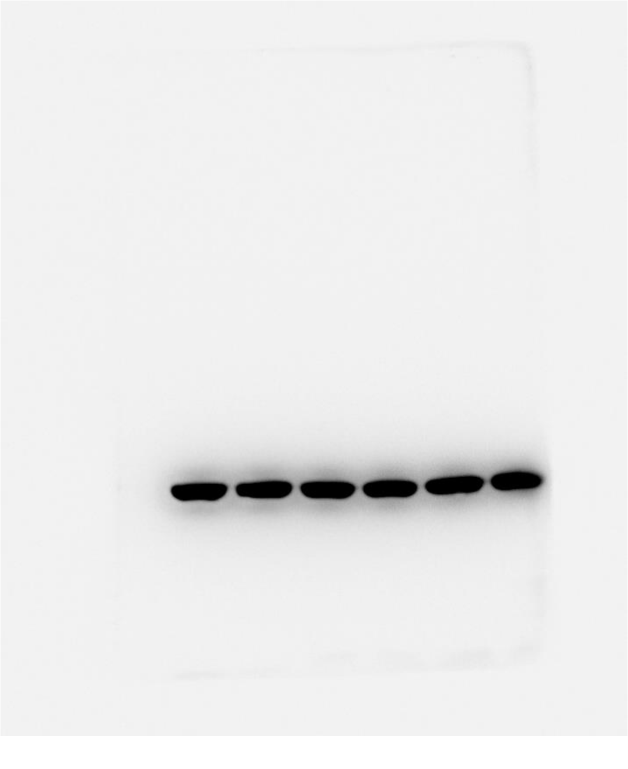

Supplementary figure 1. Effect of hyperosmolarity on HCEC viability

Sodium chloride (10mM and 100mM NaCl) was added to the HCEC culture media to induce the hyperosmolar condition. The cellular viability of the HCECs was measured using the CCK-8 reagent after 6- (A), 24- (B), 48- (C), and 72-h (D) exposure to the hyperosmolar condition. Cellular viability decreased significantly after exposure to the hyperosmolar condition compared to the control (no addition of NaCl). More than 50% of cytotoxicity was observed after 24-h exposure to the hyperosmolar culture media mixed with an additional 100mM NaCl. Triplicates of each treatment group were used in each independent experiment. The values are presented as the mean ± SEM for the four independent experiments. *** $p<0.001$

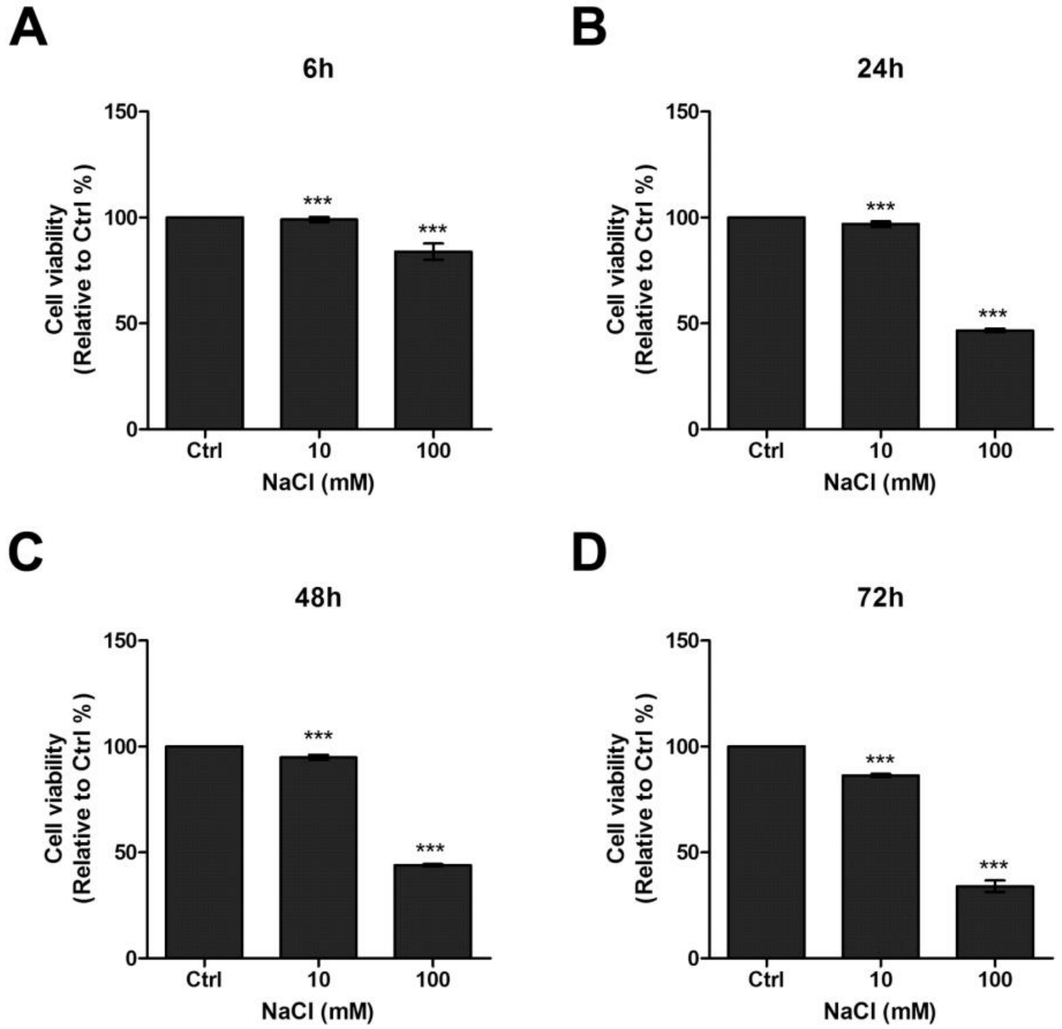

Supplementary figure 2. Induced ROS in the HCECs following exposure to the hyperosmolar condition

Sodium chloride (NaCl, 10mM and 100mM) was added to the HCEC culture media to induce the hyperosmolar condition. ROS generation from the HCECs was measured using a fluorometric intracellular ROS kit after 20-m (A), 1-h (B), and 24-h (C) exposure to the hyperosmolar condition. ROS generation significantly increased after exposure to the hyperosmolar condition. Triplicates of each treatment group were used in each independent experiment. The values are presented as the mean ± SEM for the four independent experiments. *** p<0.001

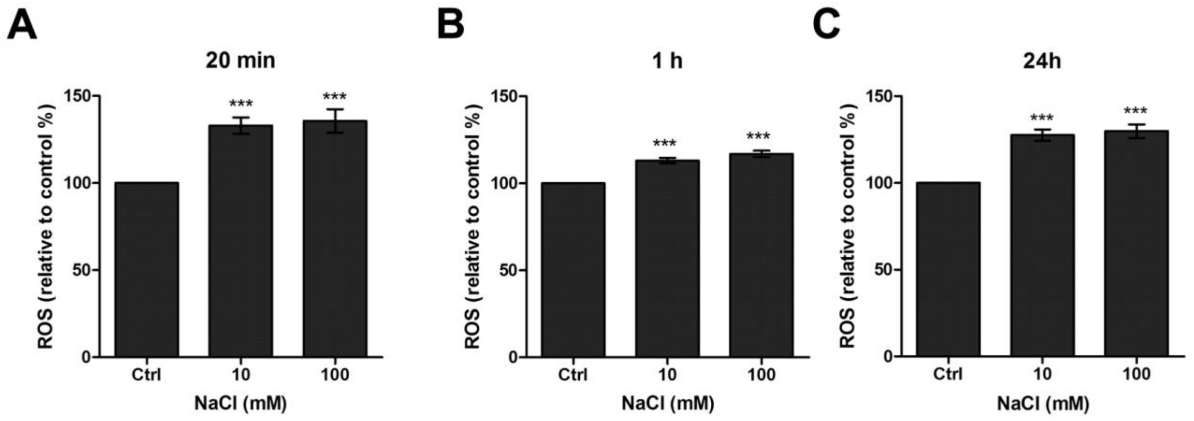

Supplementary figure 3. Representative histology images of the corneas from $NaNO_2$ treated and control (PBS treated) groups.

Mice from $NaNO_2$ treated and control (PBS treated) groups were sacrificed after 1 week of treatment. Representative hematoxylin and eosin staining images of cornea were presented. Central corneal areas (red rectangle) of panels A and C were magnified in panels B and D, respectively. Arrows indicate inflammatory cells infiltrated in corneal stroma. PBS treated cornea (A and B) shows more severe inflammation compared to $NaNO_2$ treated cornea (C and D).

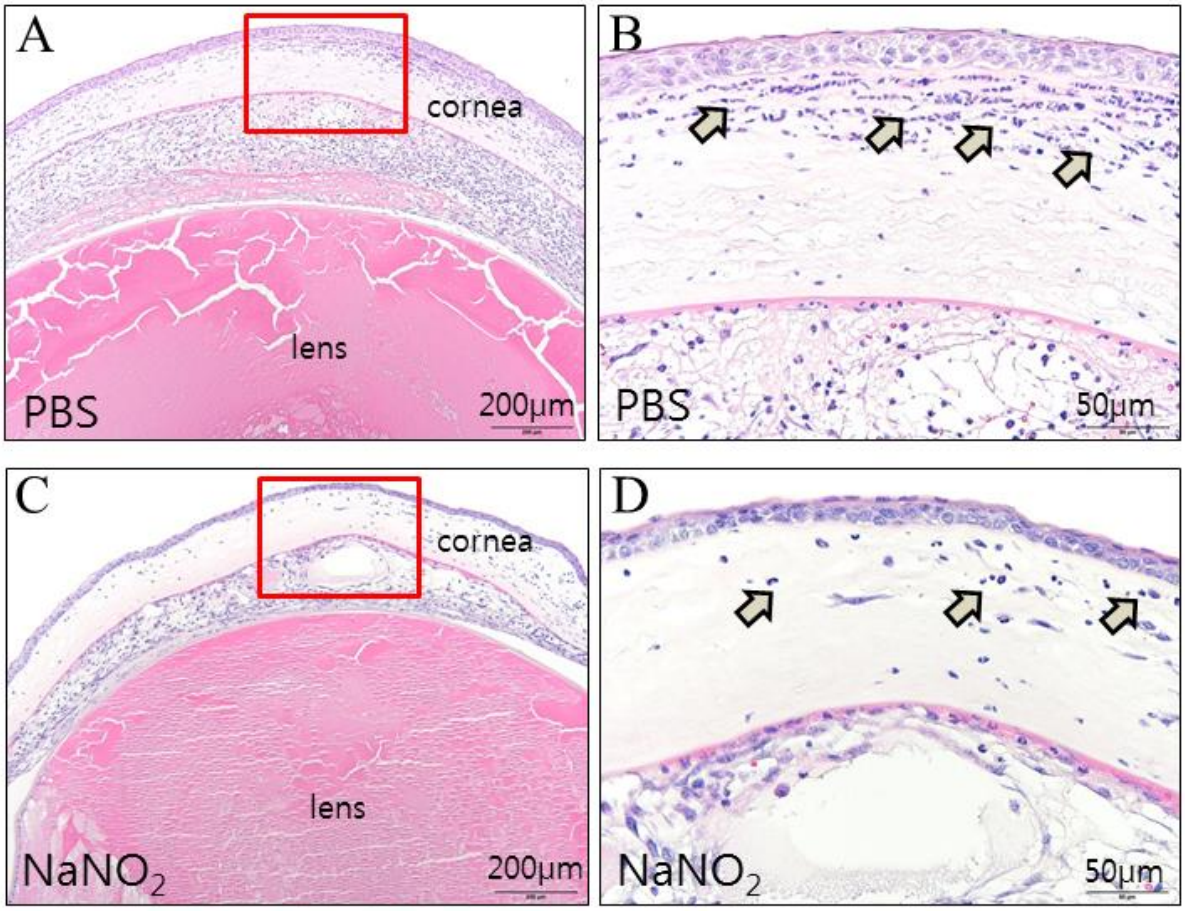

Supplement: Supplementary file 1 — Supplementary Information [file 41598_2017_8576_MOESM1_ESM.pdf]
